# Supplementary material for: Identification of photoperiod-induced specific miRNAs in the adrenal glands of Sunite sheep (Ovis aries)
Source: Front Vet Sci. 2022 Jul 22;9:888207. doi: 10.3389/fvets.2022.888207 (PMC9354845; doi:10.3389/fvets.2022.888207)
Supplement: Supplementary file 1 [file Data_Sheet_1.ZIP › Supplementary file/miRDeep2 file.zip!/miRDeep2 file/Know_Structure/SPLP42A1/oar-mir-377.pdf]

miRBase precursor : oar-mir-377
Total read count : 70
oar-miR-377-5p read count : 21
oar-miR-377-3p read count : 37
remaining reads : 12

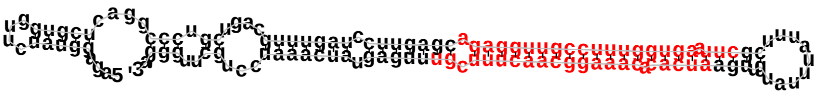

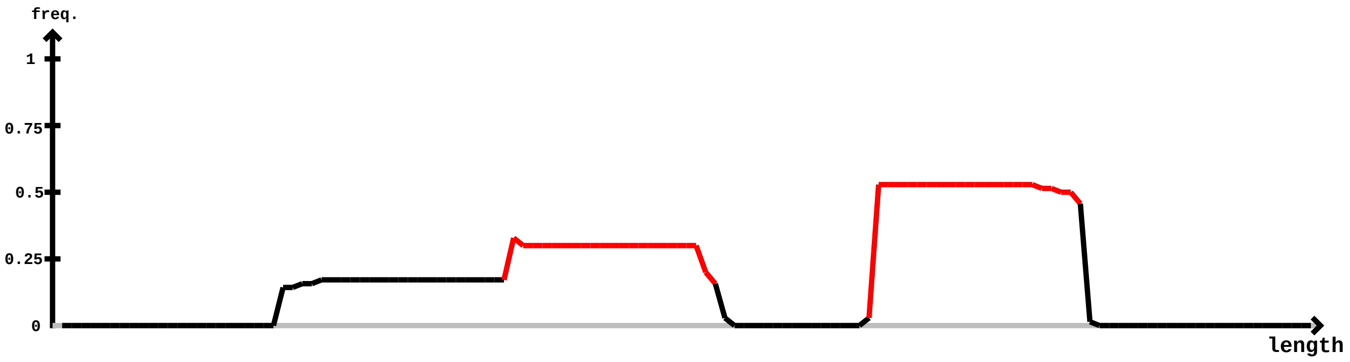


```
                                                                                  oar-miR-377-3p
                                              oar-miR-377-5p
5'- agagguaucuuggugcucaggcccugcugacguuugauccuugagcagagguugccuuuggugaauucgcuuuauuuauguugaaucacacaaaggcaacuuucguuugaguaucaaauccugcuuggga   -3'   exp
    ...(((((....)))))....(((.((....(((((((.(((((((.(((((((((((((((((.(((((.........).)))))))).))))))))))))).))))))).)))))))...))..))).   reads     mm
    ......................ccugcugacguuugauccuugagc....................................................................................   8         0
    ......................ccugcugacguuugauccuugagca...................................................................................   2         0
    ........................ugcugacguuugauccuugagc....................................................................................   1         0
    ..........................cugacguuugauccuugagc....................................................................................   1         0
    ..............................................agagguugccuuuggugaau................................................................   7         0
    ..............................................agagguugccuuuggugaauu...............................................................   3         0
    ..............................................agagguugccuuuggugaauuc..............................................................   9         0
    ..............................................agagguugccuuuggugaauucg.............................................................   2         0
    ...................................................................................aaucacacaaaggcaacuuu...........................   1         0
    ...................................................................................aaucacacaaaggcaacuuucg.........................   1         0
    ....................................................................................aucacacaaaggcaacu.............................   1         0
    ....................................................................................aucacacaaaggcaacuuucg.........................   2         0
    ....................................................................................aucacacaaaggcaacuuucgu........................   31        0
    ....................................................................................aucacacaaaggcaacuuucguu.......................   1         0
```
